# Rare Causes of Isolated and Progressive Splenic Lesions: Challenges in Differential Diagnosis, Evaluation, and Treatment of Primary Splenic Lymphomas

**DOI:** 10.1177/1179545X20926188

**Published:** 2020-06-10

**Authors:** Ryan B Sinit, Russell K Dorer, John Paul Flores, David M Aboulafia

**Affiliations:** 1 Floyd & Delores Jones Cancer Institute, Virginia Mason Medical Center, Seattle, WA, USA; 2 Department of Pathology, Virginia Mason Medical Center, Seattle, WA, USA; 3 Division of Hematology, School of Medicine, University of Washington, Seattle, WA, USA

**Keywords:** Splenic lymphoma, splenic nodules, evaluation, treatment

## Abstract

The spleen is among the most common extranodal sites for Hodgkin and non-Hodgkin lymphomas (NHLs); however, among lymphomas arising from the spleen, primary splenic lymphomas (PSLs) are rare. The group of PSLs includes primary splenic diffuse large B-cell lymphoma (PS-DLBCL), splenic red pulp small B-cell lymphoma, splenic marginal zone lymphoma (SMZL), and a splenic hairy cell leukemia variant. Delineating between the PSL variants can be challenging, especially as fine-needle aspirate and core needle biopsy of the spleen are not routinely offered at most medical centers. Herein, we describe the clinical course of 2 representative patients who presented with non-specific gastrointestinal symptoms, the first who was diagnosed with PS-DLBCL and the second who was diagnosed with SMZL. We review and contrast the clinical presentations, imaging techniques, and laboratory findings of these discrete lymphoma variants and offer strategies on how to delineate between these varied splenic processes. We also examine the use of splenectomy and splenic needle biopsy as diagnostics and, in the case of splenectomy, a therapeutic tool. Finally, we also briefly review treatment options for these varied lymphoma sub-types while acknowledging that randomized trials to guide best practices for PSLs are lacking.

## Introduction

The spleen is important in maintaining homeostasis involving the hematopoietic and phagocytic systems. As such, it is often the first organ to signal an underlying disease process. Palpable in some, splenomegaly is a common yet non-specific finding in the physical examination or radiographic imaging of patients. The causes of splenomegaly are myriad and include portal hypertension, liver disease, hematologic malignancies, infection, inflammation, and primary splenic disease.^1^ Ultrasound or computerized tomography (CT) imaging studies can reveal a solitary splenic lesion. Solid lesions of the spleen represent a heterogeneous group of diseases that include infectious, benign, and malignant etiologies (Table 1).^2^ Laboratory evaluation in conjunction with the patient’s medical and travel history are complementary in helping to identify infectious or other benign causes for splenic anomalies.

**Table 1.** Solid lesions of the spleen.

Malignant
Lymphomas
Angiosarcoma
Metastases
Sarcoma
Malignant fibrous histiocytoma (MFH)
Benign
Hamartoma
Sclerosing angiomatoid nodular transformation (SANT)
Inflammatory myofibroblastic tumor (IMT)
Extramedullary hematopoiesis (EMH)
Infectious
Tuberculosis
Fungal infection
Abscess

The spleen is among the most commonly involved extranodal sites in lymphoma; however, it is not counted as an extranodal site when calculating the revised International Prognostic Index score for non-Hodgkin lymphomas (NHLs).^3^ Splenic involvement is present in 20% of patients with an NHL and 30% to 40% of patients with Hodgkin lymphoma (HL).^4^ However, primary splenic lymphomas (PSLs) are a rare subset of B-cell NHLs that account for only 1% to 2% of all lymphomas.^5,6^ Most cases of PSL are comprised of splenic marginal zone lymphoma (SMZL) and the rest belong to an unclassified group of splenic B-cell lymphomas/leukemias—which includes primary splenic diffuse large B-cell lymphoma (PS-DLBCL), splenic red pulp small B-cell lymphoma, and a hairy cell leukemia variant.^6,7^ Distinguishing between these types can be challenging as core needle biopsy (CNB) of the spleen is presumed to be a risky strategy to secure a tissue diagnosis.

The PS-DLBCL was initially defined in 1965 as an NHL involving the spleen and hilar nodes only; others have defined it as an advanced lymphoma in which splenic involvement is the dominant feature.^8,9^ In a 2011 review, Iannitto and Tripodo sought to reconcile the disparate definitions of PS-DLBCL by distinguishing between asymptomatic patients with truly isolated splenomegaly, splenomegaly associated with alterations in peripheral blood counts, and splenomegaly associated with constitutional symptoms and abdominal discomfort.^10^ Infections with HIV, hepatitis B virus (HBV), and hepatitis C virus (HCV), often in conjunction with elevated serum lactate dehydrogenase (LDH) levels, have also been linked to PSLs.^11-14^

Herein, we describe 2 patients with increasing abdominal discomfort due to underlying PSLs of low and intermediate grade. We also briefly discuss how best to delineate between processes involving the spleen and we review contemporary treatment strategies for PSLs including surgery and chemo-immunotherapy in the era of CD20 monoclonal antibodies. Both patients have provided written informed consent for the publication of their case information and clinical images.

## Case 1

A 64-year-old Caucasian woman, whose prior medical history was significant for type 2 diabetes mellitus and hypertension, presented to medical attention in mid-2017 with intermittent left upper quadrant abdominal pain and diarrhea of several months duration. Laboratory studies included a normal chemistry panel and an unremarkable complete blood count (CBC) with the exception of a modestly elevated platelet count of 480 × 10^9^ cells/L (normal, 150-400 × 10^9^ cells/L). After 5 weeks of persistent gastrointestinal symptoms, a CT scan of the abdomen identified a hypodense splenic lesion measuring 2.7 cm and no other abnormalities. A diagnosis of sclerosing angiomatoid nodular transformation of the spleen was favored over other potential etiologies.

**Figure 1.**
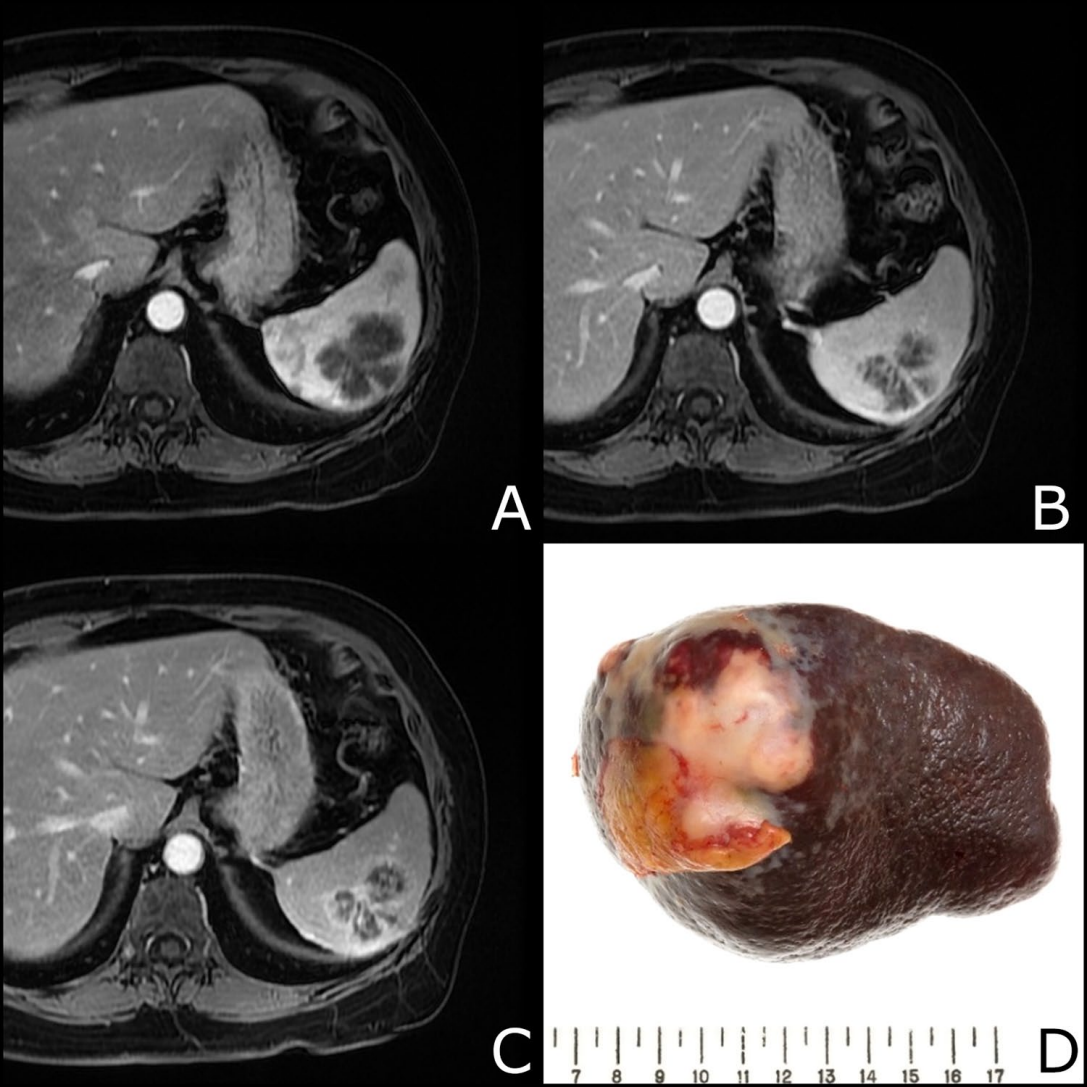
Multi-phase magnetic resonance imaging (MRI) at 1, 5, and 10 minutes (A, B, and C, respectively) showing a multi-lobulated, non-vascular 3.5 × 4.9 cm progressive heterogeneous enhancement in the spleen. (D) A section of the resected spleen showing the primary splenic diffuse large B-cell lymphoma lesion.

Five months later, she presented to medical attention complaining of intense abdominal pain, diarrhea, and 20-pound weight loss, which she attributed to increasing pain with eating as well as early satiety. She did not have fevers or night sweats. Her physical exam was notable for the absence of palpable lymphadenopathy or splenomegaly, and her CBC and comprehensive metabolic panel, including serum LDH, were all within normal limits. Her hemoglobin A1C was 6.4% (normal, 4.0%-5.6%). A CT scan of the abdomen showed left-sided colitis, no pathologically enlarged lymph nodes, and an enlarging splenic lesion. An abdominal multi-phase magnetic resonance imaging (MRI) showed the splenic lesion to be 3.5 cm × 4.9 cm in size, multi-lobulated, non-vascular, and with progressive heterogeneous enhancement in the spleen (Figure 1A-C). For her colitis, she received empiric antibiotics consisting of ciprofloxacin and metronidazole, and her abdominal discomfort gradually improved.

Given her changing radiologic findings, medical, surgical, and interventional radiology specialists were consulted to address the concern for splenic infection versus occult malignancy. She was assessed for *Toxoplasma gondii* and *Echinococcosis* infection by immunoglobulin titers and for *Mycobacterium tuberculosis* with blood cultures and Quantiferon–TB Gold release assay. C-reactive protein and erythrocyte sedimentation rate were within normal ranges. Flow cytometry (fluorescence-activated cell sorting [FACS]) analysis from peripheral blood showed no abnormal B-cell, T-cell, or natural killer (NK) cell populations. Our consultants favored laparoscopic splenectomy as both a diagnostic and therapeutic intervention, and prior to surgery, she received vaccinations for *Haemophilus influenzae* type b, meningococcal meningitis, and *Streptococcus pneumoniae*. Two months following treatment of her colitis, she underwent an uneventful splenectomy and within a few days after the operation, her persistent left upper quadrant pain had resolved.

**Figure 2.**
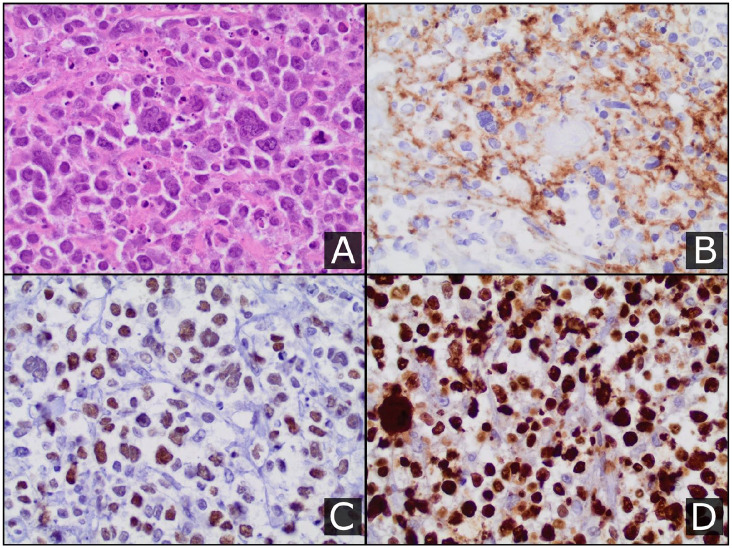
(A) Hematoxylin and eosin (H&E) staining of the solid splenic lesion showing large highly atypical lymphoid cells, including a few very large multi-nucleate and anaplastic cells. Immunohistochemical staining of the sample was positive for CD20 and BCL6 (B and C, respectively). The Ki-67 stain (D) indicated a principally 100% proliferation index in B-cells.

Examination of the spleen revealed a nearly 5 cm mass (Figure 1D). Tumor cells were positive for CD20, BCL6, MUM1, and BCL2 by immunohistochemistry and negative for AE1/AE3, CD3, and CD10. The Ki-67 proliferative index was expressed in nearly 100% of B-cell nuclei (Figure 2A to D). The FACS of the splenic tissue showed a monoclonal B-cell population. Fluorescent in situ hybridization (FISH) studies showed abnormal signaling; the *MYC* probe set showed signal fusions suggesting loss of an *MYC* locus, and the t (14; 18) probe set showed loss of the *BCL2* locus and a gain of the 5-prime *IGH* locus and no translocations involving *MYC, BCL2*, and/or *BCL6* (double or triple hit NHL). A subsequent posterior iliac crest bone marrow biopsy did not show lymphoma, and a follow-up ^18^fluorodeoxyglucose positron emission tomography/computerized tomography (^18^FDG-PET-CT) scan showed no abnormal uptake.

The patient was diagnosed with stage IE DLBCL [Stage IE refers to the Lugano classification of DLBCL (single extra-lymphatic organ/site)] for which we recommended 4 cycles of adjuvant rituximab, cyclophosphamide, doxorubicin, vincristine, and prednisone (R-CHOP) chemo-immunotherapy after hepatitis B and C serology returned non-reactive. She declined treatment recommendations, and 3 years later, she remains without evidence of lymphoma.

## Case 2

A 60-year-old Caucasian woman presented to her primary care physician in mid-2018 with abdominal bloating, persistent left upper quadrant pain, a progressive loss of appetite, occasional night sweats not characteristic of her usual hot flashes, and a loss of 27 pounds through diet. Although the weight loss was intentional, it had been consistently decreasing slowly over 8 months prior to a sudden 7-pound loss during the weeks prior to seeking medical attention. Her past medical history included arthritis of the hip and knee, basal cell carcinoma, hypercholesterolemia, seasonal allergies, celiac sprue well controlled with a gluten-free diet, and cervical spondylosis. Her physical exam was notable for a firm spleen, easily palpable in the proximal left upper quadrant, and a palpable liver. Initial laboratory studies including HIV and hepatitis B and C serology, complete metabolic panel, and LDH were unremarkable, but a CBC showed 60% lymphocytosis with a count of 19 × 10^9^ cells/L (normal, 1.00-4.50 × 10^9^ cells/L).

**Figure 3.**
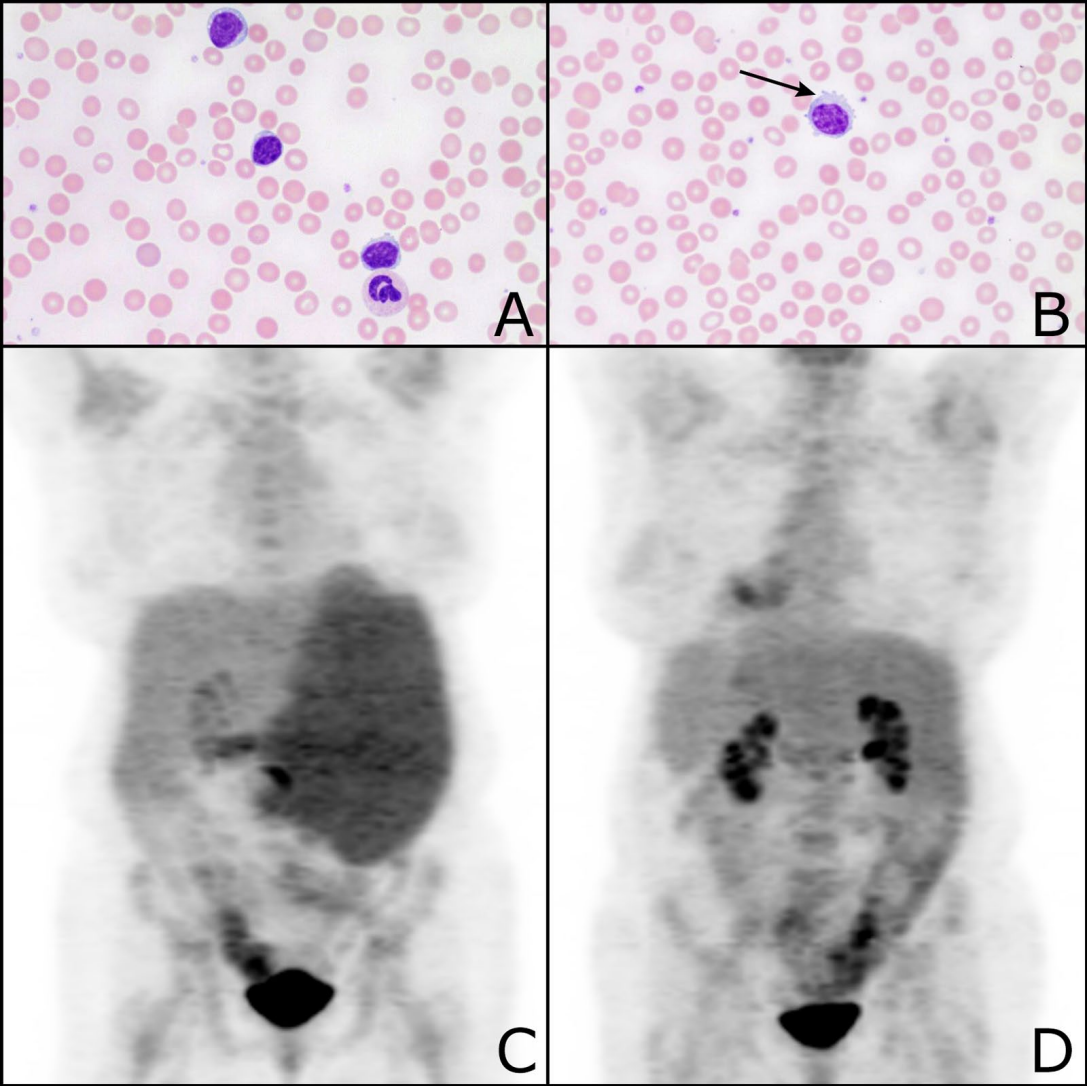
Peripheral blood smear showing lymphocytosis with (A) small mature lymphocytes and (B) occasional lymphocytes with cytoplasmic projections (arrow). (C) Whole body ^18^fluorodeoxyglucose positron emission tomography/computerized tomography (^18^FDG-PET-CT) scout film showing splenomegaly and diffuse increased uptake in the spleen. (D) Follow-up ^18^FDG-PET-CT showing complete metabolic response.

Peripheral blood showed atypical lymphocytes with occasional cytoplasmic projections (Figure 3A and B). The FACS showed leukocytosis with small B-cell lymphoproliferative disorder with an absolute B-cell count of 15.3 × 10^9^ cells/L. Cells were positive for CD20, CD22, CD23, and CD200 and negative for CD5 and CD10. A FISH panel was done to help differentiate chronic lymphocytic leukemia from marginal zone lymphoma (MZL) and was positive for 3 copies of the *MDM2* gene region (12q14) and a 6q deletion. The immunophenotypic profile favored SMZL with atypical expression of CD23.

The CT studies showed hepatosplenomegaly with the spleen measuring 22 cm in the longest dimension and para-aortic lymphadenopathy. A subsequent ^18^FDG-PET-CT showed diffusely increased metabolic activity within the spleen and para-aortic lymph nodes with a maximum standardized uptake value (SUV) of 3.4, hepatomegaly with no focal intensity, and mildly diffuse marrow activity suggestive of marrow expansion (Figure 3C).

Splenectomy was considered as the initial management strategy, but the patient preferred a less invasive approach; thus, she began rituximab 3 days after her first visit with a plan to escalate to splenectomy if she did not achieve a prompt response. She had an immediate improvement of symptoms and received 4 doses on a weekly schedule, 375 mg/m^2^. Follow-up ^18^FDG-PET-CT imaging 1 month after completion of treatment showed complete metabolic response and reduction in the spleen size to 14 cm (maximal dimension; Figure 3D).

## Discussion

Patients with solid lesions of the spleen present to medical attention with varying complaints and physical findings, the origins of which are often multiple.^15^ Physical examination of patients with unexplainable and persistent abdominal symptoms should be augmented with imaging to elucidate to what extent splenomegaly is present and to better characterize splenic lesions. Laboratory studies including FACS of peripheral blood can be normal or show non-specific findings of non-clonal leukopenia or leukocytosis. The collection of these non-diagnostic findings makes navigating the treatment labyrinth for patients with splenic lesions particularly challenging.

For patients with PS-DLBCL, splenomegaly is a common physical finding, and patients may also have abdominal pain (81%), B symptoms (59%), and impaired performance status (86%).^13,16^ Elevated serum LDH is a non-specific but common laboratory finding (84%), and reports of cytopenias have ranged between 8% and 74% in various retrospective analyses.^13,17^ The association of PS-DLBCL and HCV infection has varied considerably by region; the rate of HCV positivity in PS-DLBCL patients is 7% in Israel, 44% in Taiwan, 52% in Japan, and 64% in Italy.^12,13,18,19^ This number is unknown for the United States; however, the prevalence of HCV infection in the general US population (1.6%) is close to that of Israel (2.0%).^20^

Peripheral blood FACS as well as bone marrow aspirate and biopsy evaluations can help delineate between PSL sub-types. The PS-DLBCL involves the peripheral blood or bone marrow in less than 10% of instances, whereas other PSLs and lymphomas with secondary splenic lesions, such as were seen in case 2, more commonly involve just the spleen or both the spleen and peripheral blood or bone marrow (Table 2).^7,13,17^

**Table 2.** Hematologic malignancies that can present as solid lesions of the spleen according to the 2017 World Health Organization classification of hematopoietic and lymphoid tissues.

	Epidemiology	Clinical features	Common sites of involvement
Splenic primary			
PS-DLBCL	1% DLBCLs	Splenomegaly HCV infection High LDH B symptoms	Spleen (white pulp)
SMZL	2% lymphomas	Splenomegaly Thrombocytopenia Anemia HCV infection	Spleen Hilar lymph nodes Peripheral blood Bone marrow
Splenic red pulp small BCL	<1% NHLs	Splenomegaly Thrombocytopenia Leukopenia	Spleen (red pulp) Peripheral blood Bone marrow
HCL variant	10% HCL (2% LL)	Splenomegaly Cytopenias Leukocytosis	Spleen (red pulp) Peripheral blood Bone marrow
Non-splenic primary with primary splenic presentation			
MCL	3%-10% NHLs	Lymphadenopathy Hepatosplenomegaly	Lymph nodes Spleen Peripheral blood Bone marrow
FL	20% lymphomas	Lymphadenopathy Splenomegaly	Lymph nodes Spleen Peripheral blood Bone marrow
DLBCL, NOS	25%-30% NHLs	Dependent on involvement	Various nodal/extra-nodal sites
T-cell/histiocyte-rich large BCL	<10% DLBCLs	Fever Hepatosplenomegaly	Lymph nodes Spleen Liver Bone marrow
B-PLL	1% LL	B symptoms Splenomegaly Lymphocytosis	Spleen Peripheral blood Bone marrow
T-LGL	2%-3% mature LL	Splenomegaly Neutropenia Anemia Lymphocytosis	Spleen Liver Peripheral blood Bone marrow
Hepatosplenic TCL	<1% NHLs	Hepatosplenomegaly Thrombocytopenia Anemia Leukopenia	Spleen (red pulp) Liver Bone marrow

Abbreviations: BCL, B-cell lymphoma; B-PLL, B-cell prolymphocytic leukemia; DLBCLs, diffuse large B-cell lymphomas; FL, follicular lymphoma; HCL, hairy cell leukemia; HCV, hepatitis C virus; LDH, lactate dehydrogenase; LL, lymphocytic leukemia; MCL, mantle cell lymphoma; NHLs, non-Hodgkin lymphomas; NOS, not otherwise specified; PS-DLBCL, primary splenic diffuse large B-cell lymphoma; SMZL, splenic marginal zone lymphoma; TCL, T-cell lymphoma; T-LGL, T-cell large granular lymphocytic leukemia.

Tumors in patients with PS-DLBCL are typically confined to splenic white pulp. This is seen on diagnostic imaging as a singular or multi-focal hypodense lesion. Patients with other more indolent PSLs or secondary splenic lesions have tumors that infiltrate the entirety of the spleen or predominately the red pulp; SMZL usually presents with marked splenomegaly and lymphadenopathy, while splenic involvement of HL presents as diffuse splenic infiltration.^5^ In addition, patients with PS-DLBCL are more often diagnosed with stage I disease (42%), in contrast to patients with other types of non-splenic DLBCL for whom the incidence of stage I disease is around 28%.^21^

How best to secure a histological diagnosis of PSL is not well established and the path taken to do so often depends on institutional bias and/or provider expertise, particularly when bone marrow and FACS results are unrevealing. Biopsies of splenic lesions via CT localization or ultrasound-guided fine-needle aspirate (FNA) or CNB have traditionally not been pursued due to concerns for hemorrhagic complications. Yet, in 2 single-institution reviews which surveyed a combined total of 191 patients, only 14 (7.3%) procedures resulted in minor complications and 3 (1.5%) procedures culminated in major complications, 2 of which required splenic embolization to staunch bleeding.^22,23^ The most common minor complication was peri-splenic hematoma. Both studies reported that splenic FNA and CNB were each associated with high (>90%) sensitivity, specificity, and positive predictive values.

A histological diagnosis may continue to present challenges even after obtaining a tissue sample. Solitary splenic lesions of malignant etiology can encompass malignancies that are of true splenic origin or those that have a primary splenic presentation (Table 2). Most PSLs are germinal cell or post-germinal mature B-cell neoplasms, including PS-DLBCL, SMZL, and hairy cell leukemia.

A workflow for the evaluation of patients with isolated and non-specific splenomegaly begins with a detailed medical and travel history, physical assessment, and laboratory tests (including CBC; complete metabolic panel; LDH; serologic test for HBV, HCV, and HIV; serum protein electrophoresis [SPEP]; and serologic tests for autoimmune disorders and infections). Radiographic imaging (CT, MRI, and/or ^18^FDG-PET-CT) may also help to distinguish malignant from non-malignant splenic lesions.^10^ Due to its high sensitivity and specificity, ^18^FDG-PET-CT can be used diagnostically, as strong glucose avidity as reflected by high SUV uptake is suggestive of PS-DLBCL and is a preferred modality in the staging and follow-up of NHLs.^5^ Peripheral blood FACS and bone marrow histopathological evaluation can help delineate between the malignancies that may originate from the spleen. Depending on institutional expertise and patient comorbidities, and in the event that the etiology of the splenic abnormality remains uncertain, splenic CNB may minimize need for surgery.^22,23^

For patients with PS-DLBCL, there are no randomized clinical studies to guide treatment strategies, and specific pathways to address the workup and treatment have not been addressed specifically in the National Comprehensive Cancer Network (NCCN) guidelines. Traditionally, splenectomy has been the most common choice of physicians evaluating patients who present with masses isolated to the spleen that are suspected to be cancerous as this provides diagnostic and possibly therapeutic benefits.^13^ In a retrospective study of 87 patients with PS-DLBCL, those who underwent splenectomy not only had significantly longer progression-free survival compared with those who did not (85% vs 55%, respectively), but also they had longer 5-year overall survival (91% vs 68%, respectively).^13^ Following splenectomy, such patients will most commonly receive 4 to 6 cycles of adjuvant R-CHOP chemotherapy.^10^

Yet, the notion that all patients with PS-DLBCL require splenectomy prior to chemotherapy remains uncertain. In a retrospective analysis, 470 patients with stage I disease were stratified by whether they were diagnosed before or after the regulatory approval of rituximab in 2006.^21,24^ The analysis found that after the introduction of rituximab, the rate of splenectomy decreased from 82% to 72%. The median overall survival for patients after 2006 was 11 years compared with 9 years before 2006.^21,25,26^ An overall survival advantage with splenectomy was only seen in the pre-rituximab era (*P* = .04).^21^

For patients with low-grade PSLs, 5-year survival rates improved from 54.4% following splenectomy to 67.2% when adjuvant single-agent cytotoxic chemotherapy was also provided (*P* < .05). Five-year survival (64.7%) was not further improved if patients received adjuvant multi-agent chemotherapy after splenectomy.^21^ For patients with SMZL, the NCCN recommends the use of HCV antiviral therapy as the initial approach to patients with HCV infection and splenomegaly. If splenomegaly does not resolve after antiviral therapy, then splenectomy and adjuvant rituximab are recommended.^27^ Sustained virologic response (SVR) to HCV is possible in nearly all infected patients, and benefits associated with HCV SVR include resolution of splenomegaly and improvement in related gastrointestinal symptoms; however, the impact on lymphoma response rate is less certain.^28,29^ For patients with HCV-associated SMZL, antiviral treatment in the first-line setting was associated with a 78% 5-year progression-free survival and a 94% overall survival rate.^30^ For patients without concurrent HCV infection, NCCN guidelines recommend single-agent rituximab or splenectomy, with suggestion of using splenectomy as a salvage treatment.

For patients with PSLs, decisions regarding splenectomy should be made carefully, particularly in those with multiple or significant comorbidities. Concerns about long-term risks associated with splenectomy such as increased risk of infection, thromboembolic events, and malignancy should encourage a careful utilization of systemic and localized therapies.^31-33^ Acute complications of splenectomy include injury to the stomach or pancreas, and splenic flexure. Delayed complications can include fistulas from stomach and pancreas, sub-diaphragmatic collections, left basal atelectasis and pleural effusion, thrombocytosis and thrombosis, and overwhelming post-splenectomy infections (OPSIs), most notably from encapsulated pathogens.^6,33-35^ Infectious complications can be mitigated with appropriate vaccinations prior to splenectomy and empiric or prophylactic antibiotics, depending on clinical concerns. Our patient (case 1) with a solid splenic lesion owing to PS-DLBCL had a good outcome with laparoscopic splenectomy. In this instance, splenectomy was diagnostic and also proved to be therapeutic.

In conclusion, PSLs can be an incidental isolated finding on imaging or can be identified after workup of unexplained gastrointestinal symptoms, B symptoms, or splenomegaly. Obtaining an accurate diagnosis depends on several laboratory studies and a tissue diagnosis either by CNB if diffuse enlargement of the spleen is present, FACS, or diagnostic splenectomy. With the advent of rituximab and other CD20-targeted therapies, the need for splenectomy should be carefully considered, taking into account patient comorbidities and potential complications associated with surgery. For patients with SMZL, management is well established. In PS-DLBCL, outcomes are generally favorable. As there are currently no randomized trials for PS-DLBCL, treatment of this uncommon NHL should be regularly reviewed.
